# Utility of Pulse Wave Amplitude Drops in Assessing the Severity of Obstructive Sleep Apnea in Children and Adolescents

**DOI:** 10.1002/ppul.71223

**Published:** 2025-07-29

**Authors:** Iulia Ioan, Cyril Schweitzer, Emeline Renard, Sofia Da Mota, Laurianne Coutier, Aurore Guyon, Patricia Franco

**Affiliations:** ^1^ Service d′Explorations Fonctionnelles Pédiatriques Hôpital d′Enfants, Centre Hospitalier Universitaire de Nancy Nancy France; ^2^ DevAH, Faculté de Médecine Université de Lorraine Nancy France; ^3^ Service de Médecine Infantile Centre Hospitalier Universitaire de Nancy Nancy France; ^4^ Service de pneumologie infantile, allergologie et centre de référence en mucoviscidose Hôpital Femme Mère Enfant, Hospices Civils de Lyon Bron France; ^5^ U1028, CNRL Université de Lyon 1 Lyon France; ^6^ Service Epilepsie, Sommeil, Explorations Fonctionnelles Neurologiques Pédiatriques Hôpital Femme Mère Enfant, Hospices Civils de Lyon Bron France

**Keywords:** children, pulse wave amplitude, sleep apnea

To the Editor,

1

Obstructive sleep apnea (OSA) in children is associated with significant neurocognitive consequences, leading to poor academic performance and behavioral issues. In subjects with OSA, sympathetic activity is high throughout sleep and remains elevated during wakefulness [1]. Pulse wave amplitude drops (PWADs), measured by pulse photoplethysmography via pulse oximeter, were proposed as surrogates for these elevated sympathetic activations [2].

Children have higher arousal thresholds than adults, and respiratory events often resolve without cortical arousal [3]. However, the classical rise in sympathetic activation upon resumption of normal breathing still occurs [4], driven by afferent signals from pressure changes in the lungs, the chest wall, or the upper airways. These autonomic arousals, detected as PWADs, are thus not always associated with cortical arousals. Nonetheless, identifying PWADs may have clinical significance as they are linked to increased cardiovascular morbidity in adults [5]. In children, PWADs may contribute to sleep disturbances and neurocognitive impairments and could serve as a proxy for cortical arousal in detecting hypopneas during respiratory polygraphy.

Our objective was to determine the utility of PWADs in assessing the severity of OSA in children addressed for polysomnography (PSG).

PSG data from 91 children with median [1st; 3rd quartile] age of 10 [8; 14] years, from the Pediatric Function Testing Department of Children's University Hospital of Nancy, France, were retrospectively collected between 2022 and 2023. Nox A1 (ResMed, Reykyavik, Iceland) device with a Nonin oximeter integrated (Nonin, Plymouth, Minn, USA) was used. PSG data were manually analyzed by the sleep expert physician (II) according to the pediatric recommendations [6].

Episodes characterized by a decrease in airflow of at least 30% for at least two breaths, without desaturation or cortical arousal, were manually scored (“non‐hypopnea”). Episodes of snoring, flow limitation (FL, flattening of the inspiratory part of the flow), and PWAD (decrease of more than 30% from baseline lasting over 3 s) were automatically computed. Only FL episodes that were not part of hypopneas or non‐hypopneas were retained for analysis and reported as percentage of total respiratory cycles. PWADs were manually reviewed to remove artifacts caused by movement (Figure S1) or low oximetry signal. An event was considered associated with PWAD if it occurred concomitantly or within 5 s preceding the PWAD. A PWAD index of 50/h was considered the threshold below which sympathetic activity is poorly responsive [5]. To assess the disrupted sleep homeostasis, we computed the sleep pressure score (SPS) as previously suggested [7].

OSA was defined by an obstructive apnea‐hypopnea index (OAHI) > 1/h and categorized as mild OSA if OAHI was > 1 and < 5/h, moderate OSA if OAHI was ≥ 5 and < 10/h, and severe OSA if OAHI was ≥ 10/h.

Children and their parents were informed of the study and the pseudo‐anonymized use of their data and did not oppose. The study was approved by the ethics committee of the University Hospital of Nancy, France, registered with the number 2024PI033, and recorded on clinicaltrials.gov with the number NCT06754800.

Statistical analysis was performed with the SAS OnDemand for Academics software. Data were expressed as median [1st; 3rd quartile] for quantitative variables and as number (percentage) for qualitative variables. Comparisons were performed using paired samples non‐parametric Wilcoxon test, non‐parametric Kruskal–Wallis ANOVA, and Tukey–Kramer procedure or Fisher exact test as needed. After Bonferroni correction, *p* ≤ *0.004* was considered statistically significant. Spearman's rank correlation test was used to assess the relationships between variables and a *p* ≤ *0.05* was considered statistically significant.

Of the 91 children analyzed, 31 (34%) presented with non‐comorbid OSA. 60 (66%) had comorbidities as history of prematurity (*n* = 2), anomalies of upper airways (*n* = 1), neurological (*n* = 4), cardio‐respiratory (*n* = 9), metabolic (*n* = 31), genetic (*n* = 9), or hematological disorders (*n* = 2). All children were of Caucasian ethnicity except one, who was African‐Caribbean. PSG results showed that OAHI, central apnea index and non‐hypopnea index were significantly higher in REM (rapid‐eye movement) sleep than in non‐REM (slow‐wave) sleep (*p* < *0.0001*, Table 1), with no significant difference for the percentage of FL or the snoring index. All PWAD indexes were significantly higher in REM than in non‐REM sleep (*p* ≤ *0.0005*), with no difference for the PWAD index related to obstructive apnea or snoring. After adjusting for the total amount of REM or NREM sleep, the total PWAD index was significantly higher in NREM than in REM sleep (*p* < *0.0001*).

**Table 1:** Polysomnography data in the entire cohort of children and the paired comparison between the respiratory parameters obtained in REM (rapid‐eye movement) sleep and in non‐REM (slow‐wave) sleep.

	TST	Non‐REM sleep	REM sleep
*n*	91	91	91
Total sleep time, min	432 [383; 501]	362 [324; 404]	80 [57; 101]
Obstructive apnea‐hypopnea index	5.4 [3.6; 7.7]	3.7 [2.4; 7.2]	10.8 [5.2; 16.2]*
Central apnea index	0.6 [0.2; 1.3]	0.3 [0; 0.9]	1.1 [0; 2.6]*
Non‐hypopnea index	13.5 [9.9; 18]	8.4 [5.1; 13.2]	34.6 [23.8; 45]*
Flow limitation, % of TST	15.7 [8.5; 22.2]	15.4 [7.8; 22.8]	15.9 [9.8; 23.8]
Snoring index	80 [29; 236]	80 [21; 241]	63 [13; 213]
Total PWAD index	55 [39; 71]	51 [35; 68]	70 [47; 86]*
Total PWAD index adjusted		42 [30; 59]	11 [7.0; 16]*
PWAD index related to CA	0.3 [0; 0.9]	0.1 [0; 0.6]	0.6 [0; 2]
PWAD index related to OA	0.2 [0; 0.6]	0.2 [0; 0.5]	0 [0; 0.7]
PWAD index related to hypopneas	2.7 [1.4; 4.3]	1.9 [0.8; 3.4]	5.4 [2.1; 10.9]*
PWAD index related to non‐hypopneas	8.3 [5; 12.1]	4.6 [2.7; 7.8]	23.6 [12.5; 32.1]*
PWAD index related to FL	23.6 [14.2; 36.2]	20.5 [10.8; 32.2]	33.1 [18.9; 47.3]*
PWAD index related to snoring	9.9 [4.5; 21.8]	10.1 [4.4; 17.1]	9.1 [3.6; 26.3]
PWAD index related to cortical arousals	4.7 [2.7; 6.7]	4.3 [2.7; 6.6]	5.8 [2.3; 9.8]**
PWAD index related to leg movements	5.2 [2.4; 9.4]	4.6 [2.2; 9.1]	6.4 [2; 14.7]***

*Note:* Data are expressed as median [1st; 3rd quartile].

Abbreviations: CA = central apnea; FL = flow limitation; non‐hypopnea = decrease in airflow signal of at least 30% for at least two breaths without desaturation or cortical arousal; OA = obstructive apnea; PWAD = pulse wave amplitude drop; TST = total sleep time.

* *p* < 0.0001

** *p* = 0.0003

*** *p* = 0.0005.

Of the total PWAD index, the PWAD index related to FL had the highest proportion (50 [33; 59]%), followed by snoring (21 [9; 42]%), non‐hypopneas (17 [11; 23]%), leg movements (10.8 [5.6; 21]%) and cortical arousals (8.8 [5.9; 15.1]%). The PWAD index related to hypopneas accounted for 5.5 [3.3; 9.1]%, while that related to obstructive apnea 0.4 [0; 1.1]% and to central apnea 0.6 [0; 1.6]%. Among all children, 40 (44%) had mild OSA, 35 (38%) had moderate OSA, and 16 (18%) had severe OSA (Table 2). In severe OSA, total and respiratory‐related arousal indexes, as well as SPS, the hypopnea and snoring indexes, were significantly higher (*p* < *0.0001* for all) compared to moderate and mild OSA. In contrast, the proportion of FL episodes was lower, without reaching statistical significance. Total PWAD index and the PWAD indexes related to non‐hypopneas and FL were significantly lower during REM sleep in severe OSA compared to mild or moderate OSA (*p* < *0.0001*), with a similar trend found during TST and non‐REM sleep. PWAD indexes related to hypopneas (during TST, non‐REM, and REM sleep) and to snoring (during TST and REM sleep, but not non‐REM sleep) were significantly higher in severe OSA compared to mild or moderate OSA (*p* < *0.0001* for all).

**Table 2:** Comparison between the pulse wave amplitude drop (PWAD) indexes, computed on total sleep time and separately in REM (rapid‐eye movement) sleep and non‐REM (slow‐wave) sleep, among the three severity categories of obstructive sleep apnea (OSA).

	Mild OSA		Moderate OSA		Severe OSA	
	Non‐REM sleep	REM sleep	Non‐REM sleep	REM sleep	Non‐REM sleep	REM sleep
*n*	40		35		16	
Age	10.2 [7.5; 13.6]		9.7 [7.4; 12.8]		11.7 [8.7; 14.7]	
Total arousal index	8.2 [6.2; 10.1]		10.9 [6.9; 13.4]		19.7 [12.7; 25.9]*	
Respiratory‐related arousal index	2.1 [1.5; 3.0]		3.4 [2.3; 4.4]		8.9 [6.1; 14.4]*	
Spontaneous arousal index	3.0 [2.2; 5.5]		3.5 [2.6; 5.4]		3.6 [2.4; 5.8]	
SPS (sleep pressure score)	0.16 [0.08; 0.21]		0.22 [0.15; 0.28]		0.42 [0.23; 0.52]*	
Snoring index	37 [15; 100]		80 [32; 168]		285 [189; 410]*	
Flow limitation (%)	13.9 [7.0; 18.2]		17.7 [9.5; 27.9]		15.7 [7.3; 25.9]	
Hypopnea index	2.6 [1.5; 3.4]		5.1 [4.5; 6.2]**		14.4 [11.7; 27.2]*	
OAHI	3.4 [2.6; 4.2]		6.1 [5.4; 7.3]**		15.0 [12.8; 27.8]*	
	2.5 [1.6; 3.2]	6.9 [3.6; 10.8]	5.3 [3.6; 7.0]	12.2 [7.8; 16.0]	13.7 [9.7; 23.8]	28.7 [22.1; 47.3]
Total PWAD index	56.1 [36.1; 69.6]		56.4 [43.5; 74.2]		41.2 [25.1; 66.2]	
	53.3 [32.6; 65.1]	71.3 [48.4; 90.3]	52.6 [42.0; 73.7]	76.8 [48.4; 86.7]	38.2 [23.7; 67.3]	49.3 [38.5; 59.6]*
PWAD index related to CA	0.2 [0.1; 0.9]		0.4 [0; 0.9]		0.2 [0; 0.8]	
	0 [0; 0.6]	0.9 [0; 2.3]	0.2 [0; 0.6]	0 [0; 2.0]	0.2 [0; 0.6]	0 [0; 1.6]
PWAD index related to OA	0.1 [0; 0.6]		0.3 [0; 0.7]		0.3 [0; 0.8]	
	0 [0; 0.5]	0 [0; 0.7]	0.3 [0; 0.5]	0 [0; 1.1]	0.3 [0; 0.8]	0
PWAD index related to hypopneas	1.6 [0.8; 2.6]		3.3 [2.6; 4.4]**		6.0 [4.1; 8.7]*	
	1.0 [0.5; 1.7]	3.5 [1.1; 7.4]	2.6 [1.5; 3.6]**	6.6 [3.5; 11.1]	5.4 [2.7; 7.8]*	12.1 [6.9; 15.5]*
PWAD index related to non‐hypopneas	8.2 [5.5; 11.7]		10.4 [5.9; 12.5]		5.6 [3.5; 8.5]	
	4.1 [2.6; 7.8]	24.9 [16.8; 31.8]	5.3 [3.4; 8.1]	30.2 [17.1; 36.0]	4.5 [2.0; 6.8]	8.9 [4.2; 14.8]*
PWAD index related to FL	19.8 [13.9; 31.1]		28.6 [17.6; 38.5]		14.9 [5.6; 31.3]	
	16.3 [10.5; 28.2]	37.6 [20.7; 49.6]	28.5 [16.4; 37.9]	37.0 [25.9; 53.1]	13.5 [4.9; 31.8]	16.7 [9.1; 26.1]*
PWAD index related to snoring	6.6 [3.8; 13.5]		11.6 [5.7; 21.9]		20.6 [8.3; 36.3]*	
	6.6 [3.6; 13.1]	5.4 [3.1; 16.5]	11.0 [5.3; 16.8]	13.3 [4.1; 30.3]	20.4 [8.1; 36.4]	10.1 [3.4; 33.6]
PWAD index related to cortical arousals	4.4 [2.3; 6.2]		5.2 [3.7; 6.9]		5.0 [2.4; 6.2]	
	3.4 [2.2; 6.2]	5.6 [2.4; 9.9]	4.7 [4.0; 6.8]	6.1 [3.2; 9.4]	4.2 [2.8; 6.9]	3.0 [0.8; 10.7]
PWAD index related to leg movements	5.4 [2.9; 11.8]		5.3 [2.1; 9.3]		3.8 [1.7; 8.1]	
	4.4 [2.5; 9.5]	8.5 [2.7; 15.6]	4.6 [1.9; 9.7]	5.0 [1.6; 12.4]	4.3 [1.4; 7.3]	4.1 [0; 12.2]

*Note:* Data are expressed as median [1st; 3rd quartile]. Abbreviations as in Table 1.

* *p* < 0.0001: severe OSA compared to mild or moderate OSA

** *p* < 0.0001: moderate OSA compared to mild OSA.

No significant differences were found in PWAD indexes related to obstructive or central apneas, cortical arousals, or leg movements, across OSA severity levels.

A higher proportion of children with severe OSA, 11 (69%), had a PWAD index below 50/h compared to those with moderate OSA, 12 (34%), (*p* = 0.02), or with mild *OSA*, 16 (40%) (*p* = 0.08).

SPS showed a positive correlation with OAHI (rho = 0.55, *p* < *0.0001*), hypopnea index (rho = 0.45, *p* < *0.0001*), total (rho = 0.28, *p* < *0.008*) and respiratory‐related cortical arousal index (rho = 0.70, *p* < *0.0001*) and snoring index (rho = 0.31, *p* = *0.003*), and a negative correlation with spontaneous arousal index (rho = −0.49, *p* < *0.0001*). Moreover, SPS was significantly correlated with the PWAD index related to hypopneas (rho = 0.33, *p* = *0.001*) and to snoring (rho = 0.25, *p* = *0.015*).

In the present study, among children with OSA, the PWAD indexes were higher during REM than during non‐REM sleep, with the PWAD index related to FL representing the largest proportion of the total PWAD index, followed by the PWAD index related to snoring, and the PWAD index related to hypopneas without desaturations or cortical arousals. Children with severe OSA had significantly higher PWAD indexes related to hypopneas and snoring, and lower total PWAD indexes and PWAD indexes related to non‐hypopneas and FL in comparison to children with mild or moderate OSA.

Latest studies in adults have challenged the use of the OAHI, a metric measure, for guiding treatment decision as it does not reflect cardiovascular and cognitive morbidity. Children with mild OSA [8] and those who snore [9] have worse results on neurocognitive and behavioral assessments compared to children who do not snore. In these domains, even after controlling for the baseline variables and OAHI severity, snoring status emerged as a significant predictive factor, while OAHI did not significantly predict the overall cognitive performance [9]. Additional parameters are required to promptly identify the existence of abnormal sleep‐related breathing to avert its repercussions. The significant proportion of PWAD index related to FL or snoring reported in our study suggests that these respiratory events warrant attention, and these children require monitoring and treatment, as previously underlined that respiratory efforts trigger arousals from sleep, daytime fatigue, or sleepiness [10]. This is further confirmed by the positive correlation between SPS, a measure of disrupted sleep [7], and PWAD index related to hypopneas or snoring. Atypical respiratory patterns during sleep (i.e. FL, mouth‐breathing, changes in inspiratory and expiratory time, rib cage and expiratory muscle activity, or snoring noises) should be thoroughly assessed as contributing to sleep disturbances. Moreover, these abnormal respiratory patterns were shown to resolve with the appropriate application of continuous positive airway pressure. A comprehensive analysis of EEG using Fast Fourier Transformation indicated a change in the sleep microstructure in children with FL compared to those without FL, not identified by the human eye. This change implied an increase in slow delta band as a persistent effort to maintain sleep and counteract its disruption, and an abnormal reduction in theta and increase in alpha rhythms as an ongoing disturbance in sleep [11]. These small alterations not seen on electroencephalogram might be identified by other parameters as PWAD.

Children with mild or moderate OSA had higher proportions of FL episodes and PWAD indexes related to non‐hypopneas and FL compared to those with severe OSA. This further suggests that, in severe OSA, these patterns were likely replaced by actual hypopneas, highlighting the importance of recognizing them, particularly in less severe cases, as early intervention may help prevent neurocognitive complications [12, 13].

Children with severe OSA had higher PWAD indexes related to hypopneas and snoring, as well as higher SPS compared to moderate or mild OSA. Moreover, a higher proportion exhibited a PWAD index below 50/h, indicating a poorly responding autonomic nervous system. In adults with severe OSA, it has been proposed that a blunted sympathetic response leads to baroreflex tolerance, which correlates with older age and elevated blood pressure [14]. We wonder if the children with low PWAD indexes, thus low autonomic response, might be at risk of neurocognitive and cardiovascular impairment compared to children with higher PWAD indexes. Future studies are needed to evaluate the neurocognitive impact of a blunted autonomic response.

Our study has several limitations. The retrospective design precluded the assessment of clinical symptoms and treatment outcomes in these children. An automated approach was chosen to provide clinicians with a readily accessible tool, supplemented by manual review of PWAD events to enhance analysis sensitivity. Our major objective was to establish PWADs as a viable alternative to cortical arousals for evaluating non‐desaturating hypopneas, which could be useful in the context of respiratory polygraphy.

In conclusion, in our cohort of children and adolescents with OSA and comorbidities in most of them, the PWAD index related to FL represented the most important part of the total PWAD index, and children with severe OSA had lower total PWAD indexes and higher PWAD indexes related to hypopneas and snoring. Our findings suggest that in children with mild and moderate OSA, PWADs associated with abnormal respiratory patterns should be taken into account when considering treatment strategies, as they may serve as early indicators of disease progression. In contrast, a low PWAD index in children with severe OSA should prompt further investigation in larger cohorts, as it may be linked to neurocognitive development and cardiovascular morbidity, possibly reflecting a more chronic or impactful form of the OSA disease.

## Author Contributions

**Iulia Ioan:** conceptualization, investigation, funding acquisition, writing – original draft, methodology, validation, writing – review and editing, software, formal analysis, project administration. **Cyril Schweitzer:** funding acquisition, writing – original draft, writing – review and editing, supervision, methodology, validation, formal analysis. **Emeline Renard:** investigation, writing – original draft, validation, methodology, formal analysis, writing – review and editing. **Sofia Da Mota:** investigation, writing – original draft, methodology, validation, formal analysis, writing – review and editing. **Laurianne Coutier:** conceptualization, investigation, writing – original draft, writing – review and editing, methodology, formal analysis. **Aurore Guyon:** conceptualization, writing – original draft, writing – review and editing, validation, methodology, formal analysis, software. **Patricia Franco:** supervision, conceptualization, writing – original draft, writing – review and editing, project administration, methodology, validation, formal analysis, software.

## Conflicts of Interest

The authors declare no conflicts of interest.

## Supporting information


Figure S1: Screenshot of a 2‐minute recording of polysomnography of an 11 years old boy.

PSG comprises neurophysiological signals (two electrooculograms, electroencephalogram by two frontal, two central, and two occipital leads, and two electromyograms by submental leads), as well as respiratory signals (snoring volume by microphone, nasal flow, calibrated flow‐RIP, thoraco‐abdominal efforts measured by thoraco‐abdominal belts, pulse plethysmography, oxygen saturation and cardiac frequency via pulse oximetry, activity and body position assessed by an actimetry sensor). On the plethysmography signal, the flash indicates a manually scored artifact that replaced a PWAD previously detected automatically by the software. Additionally, two other PWADs were automatically detected, one before and one after the artifact.

## Data Availability

The data that support the findings of this study are available from the corresponding author upon reasonable request.
