# Supplementary figures and images for: Utility of Pulse Wave Amplitude Drops in Assessing the Severity of Obstructive Sleep Apnea in Children and Adolescents
Source: Pediatr Pulmonol. 2025 Jul 29;60(7):e71223. doi: 10.1002/ppul.71223 (PMC12308168; doi:10.1002/ppul.71223)

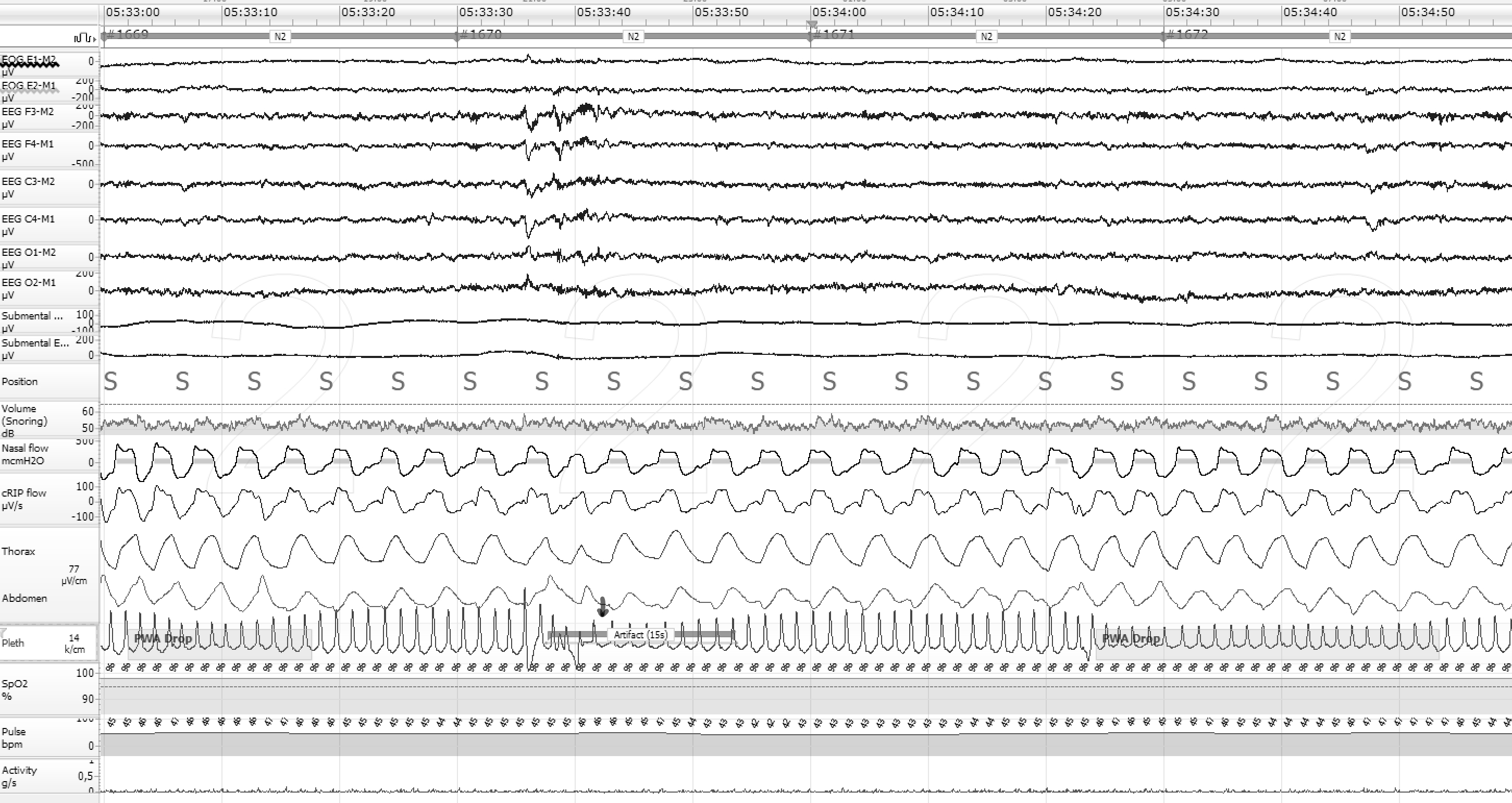

Supplement: Supplementary file 1 — Figure S1: Screenshot of a 2‐minute recording of polysomnography of an 11 years old boy. [file PPUL-60-0-s001.tif]
