# Supplementary material for: Utility of Pulse Wave Amplitude Drops in Assessing the Severity of Obstructive Sleep Apnea in Children and Adolescents
Source: Pediatr Pulmonol. 2025 Jul 29;60(7):e71223. doi: 10.1002/ppul.71223 (PMC12308168; doi:10.1002/ppul.71223)
Supplement: Supplementary file 2 — PSG comprises neurophysiological signals (two electrooculograms, electroencephalogram by two frontal, two central, and two occipital leads, and two electromyograms by submental leads), as well as respiratory signals (snoring volume by microphone, nasal flow, calibrated flow‐RIP, thoraco‐abdominal efforts measured by thoraco‐abdominal belts, pulse plethysmography, oxygen saturation and cardiac frequency via pulse oximetry, activity and body position assessed by an actimetry sensor). On the plethysmography signal, the flash indicates a manually scored artifact that replaced a PWAD previously detected automatically by the software. Additionally, two other PWADs were automatically detected, one before and one after the artifact. [file PPUL-60-0-s002.docx]

Figure 1 suppl.

**Screenshot of a 2-minute recording of polysomnography of an 11 years old boy.** PSG comprises neurophysiological signals (two electrooculograms, electroencephalogram by two frontal, two central, and two occipital leads, and two electromyograms by submental leads), as well as respiratory signals (snoring volume by microphone, nasal flow, calibrated flow-RIP, thoraco-abdominal efforts measured by thoraco-abdominal belts, pulse plethysmography, oxygen saturation and cardiac frequency via pulse oximetry, activity and body position assessed by an actimetry sensor).

On the plethysmography signal, the flash indicates a manually scored artifact that replaced a PWAD previously detected automatically by the software. Additionally, two other PWADs were automatically detected, one before and one after the artifact.

Online references

E1. Somers VK, Dyken ME, Clary MP, Abboud FM. Sympathetic neural mechanisms in obstructive sleep apnea. *The Journal of clinical investigation.* 1995;96(4):1897-1904.

E2. Colombo R, Marchi A, Borghi B, et al. Pulse Photoplethysmographic Analysis Estimates the Sympathetic Activity Directed to Heart and Vessels. *Anesthesiology.* 2015;123(2):336-345.

E3. Lopes MC, Marcus CL. The significance of ASDA arousals in children. *Sleep medicine.* 2007;9(1):3-8.

E4. Berry RB, Budhiraja R, Gottlieb DJ, et al. Rules for scoring respiratory events in sleep: update of the 2007 AASM Manual for the Scoring of Sleep and Associated Events. Deliberations of the Sleep Apnea Definitions Task Force of the American Academy of Sleep Medicine. *Journal of clinical sleep medicine: JCSM: official publication of the American* *Academy of Sleep Medicine.* 2012;8(5):597-619.

E5. Tauman R, O’Brien LM, Holbrook CR, Gozal D. Sleep pressure score: a new index of sleep disruption in snoring children. Sleep. 2004 Mar 15;27(2):274-8.

☑E6. Hirotsu C, Betta M, Bernardi G, et al. Pulse wave amplitude drops during sleep: clinical significance and characteristics in a general population sample. *Sleep.* 2020;43(7).

E7. Smith DL, Gozal D, Hunter SJ, Kheirandish-Gozal L. Frequency of snoring, rather than apnea-hypopnea index, predicts both cognitive and behavioral problems in young children. Sleep Med. 2017 Jun:34:170-178

☑E8. Gozal D, Kheirandish L. Oxidant stress and inflammation in the snoring child: confluent pathways to upper airway pathogenesis and end-organ morbidity. Sleep Med Rev. 2006 Apr;10(2):83-96.
